# Editorial: Endocrine regulation of mineral ions and their relevance to metabolic diseases

**DOI:** 10.3389/fendo.2024.1391096

**Published:** 2024-04-03

**Authors:** Mor-Li Hartman, Mohammed S. Razzaque

**Affiliations:** ^1^ Department of Inflammation and Immunology, ADA Forsyth Institute, Cambridge, MA, United States; ^2^ Department of Medical Education, School of Medicine, University of Texas Rio Grande Valley (UTRGV), Edinburg, TX, United States

**Keywords:** endocrine regulation, minerals ion, metabolic diseases, metabolic homeostasis, health

One of the significant global challenges over the last couple of decades has been the emergence of the metabolic syndrome, a major global health concern (1). Extensive research is being conducted to understand and address this issue, with a particular focus on the role of minerals in metabolic diseases. Essential nutrients and minerals such as phosphate, calcium, magnesium, zinc, selenium, and iron, along with vitamins, play a vital role in maintaining the body’s metabolic balance. There is a growing body of research aimed at better comprehending the significance of minerals and their levels in the body, especially concerning metabolic syndrome (2–5). Previous study by Hartman et al. have indicated that salivary phosphate levels can potentially predict the onset of obesity in children (6). Despite advancements in understanding the underlying mechanisms of metabolic diseases, there are still gaps in knowledge regarding how imbalances in various minerals and nutrients contribute to the development and progression of metabolic disorders.

It is well-documented that minerals and trace elements are essential micronutrients with well-defined biochemical and biological functions. Inadequacy in these micronutrients is associated with widespread human health issues. For instance, some diabetic patients may have deficiencies in zinc, chromium, and magnesium, highlighting the importance of these minerals in health and disease (7).

Magnesium is an essential nutrient that plays a crucial role in various physiological functions in the body (8, 9). It has been associated with a protective role in metabolic disorders, particularly in relation to the activation of vitamin D and its impact on immune function (10, 11). The paper by Eskander and Razzaque provided evidence that magnesium may help reduce the severity of asthma symptoms, reduce the intensity of COVID-19 symptoms, and inflammation. Additionally, there is evidence suggesting that magnesium is involved in improving glucose and insulin metabolism, making it relevant to conditions such as obesity, metabolic syndrome, and type 2 diabetes (12, 13). However, while the findings offer valuable insights, further research, including randomized controlled trials, is needed to validate these associations and determine causality with potential impact on vaccination and related adverse events (14). The significance of vitamin D and its impact on healthy glucose metabolism is an ongoing area of research, particularly in relation to magnesium. Zittermann et al. described the potential role of magnesium in the synthesis of vitamin D (15). Gong et al. concluded that the level of vitamin D is independently correlated with the HOMA-β index and pancreatic β-cell dysfunction, and that magnesium intake enhances this association. However, the cross-sectional nature of the study prevents the determination of causality. Liu et al. found an independent association between the level of vitamin D and insulin resistance, which was modified by different levels of magnesium intake. The research suggests that optimal magnesium status may be important for optimizing vitamin D status (16, 17), and that the interaction between magnesium and vitamin D has significant implications for metabolic health.

Liu et al. conducted a study to examine the association between different levels of vitamin D and metabolic health indicators. The research focused on the relationship between triglycerides/high-density lipoprotein (TG/HDL) ratio with vitamin D levels and insulin resistance, impaired glucose tolerance, and diabetes mellitus. Using a cross-sectional analysis of the American National Health and Nutrition Examination Survey (NHANES) database, the authors found that both too-low and too-high levels of vitamin D3 can support the association of TG/HDL as a risk factor for Insulin resistance, impaired glucose tolerance, and diabetes mellitus. They concluded that maintaining vitamin D levels within an optimal range is essential to delay the progression of metabolic health issues such as insulin resistance, impaired glucose tolerance, and type 2 diabetes. The research has also indicated a connection between obesity and vitamin D deficiency, as well as low calcium levels (18). Yang et al. suggested that exposure to famine may be a factor contributing to changes in circulating calcium concentrations. They proposed that establishing a normal range of serum calcium and considering famine exposure history, especially in females, could help identify individuals with abnormal calcium levels and related diseases early. On the other hand, Li et al. proposed that low serum calcium could contribute to retinopathy in non-diabetic individuals. They suggested that maintaining a higher serum calcium level may be recommended to reduce the development of retinopathy.

The association between high phosphate levels and glucose impairment has been a subject of research (4, 19). This line of investigation originated from the observation of elevated phosphate levels in the saliva of obese children in Kuwait (6, 20). This led to the exploration of the potential link between obesity and phosphorus, and why high phosphate levels are found in obese children. Alhareky et al. conducted a study examining the beverage consumption of children in Kuwait, which revealed that those with high soda consumption had a significantly higher prevalence of obesity. While high obesity prevalence was also observed with high milk consumption, it was not as significant, and no such association was found with high juice consumption. The investigators discussed the possibility that the phosphate from soda drinks, in combination with sugar, may contribute to the development of obesity. The study sheds light on the potential impact of beverage choices on obesity and the need for additional studies to validate these findings. Using the same Kuwaiti cohort, a study published in 2016 found a significant association between dental decay and calorie-adjusted sugar intake. Interestingly, the study also revealed a significantly high percentage of dental decay in children who consumed a diet low in sugar but high in phosphate, compared to those who consumed a diet low in both sugar and phosphate (21). This result highlights the potential impact of both high sugar and high phosphate consumption on dental health (21, 22). Hetz et al. took a distinct approach to investigating the effects of excessive phosphate on subcellular cell signaling. They utilized quantitative proteomics and phosphoproteomics to explore the role of inorganic phosphate in protein expression and phosphorylation modification. The researchers also conducted bioinformatic analyses and literature reviews, revealing that elevated inorganic phosphate levels can rewire cell signaling through extensive cross talk. Additionally, their use of western blot analysis confirmed significant changes in the regulators responsible for pre-mRNA alternative splicing. The study sheds light on the potential impact of high phosphate intake on cellular processes and the need for further research to fully understand its consequences (23, 24).

The studies have elucidated the complex mechanisms involved in regulating phosphate homeostasis and its associated cytotoxicity. The kidney plays a critical role in maintaining the balance of mineral ions, such as calcium, phosphate, zinc, and magnesium, in the body, through processes like renal excretion, tubular reabsorption, and fine adjustments of urinary excretion to maintain the balance related to net intake. Imbalances in these ions can lead to unwanted clinical complications, and the kidney’s role in regulating their balance is of great physiologic importance (25–28).

The article by Michigami et al. provides an overview of the role of osteocytes in phosphate metabolism, focusing on their function as dendritic cells in mineralized bone. The paper discusses how osteocytes control bone mass by producing sclerostin, an inhibitor of bone formation, and receptor activator of nuclear factor κ B ligand, an inducer of osteoblastic bone resorption. Additionally, it highlights the role of osteocytes in governing phosphate homeostasis through the production of fibroblast growth factor 23 (29). The article also delves into the molecular mechanisms through which osteocytes regulate phosphate metabolism, shedding light on their critical role in growth-related alterations and phosphate sensing in the body.

The study by Mohammadifard et al. provides a systematic review of randomized clinical trials, summarizing the impact of consuming whole soy, soy products, and its isolated components on metabolic syndrome features in adults. The research revealed that the inclusion of soy products in the diet of patients with metabolic syndrome effectively improved lipid profile and glycemic parameters, independent of its impact on anthropometric measures (30). In a separate study, Yan et al. aimed to determine the role of soy intake in the risk of type 2 diabetes. The findings indicated that soy intake from tofu was inversely associated with the risk of diabetes among women, with the inverse association being more pronounced among overweight and postmenopausal women, but not among men.

These studies underscore the potential benefits of soy and its products in improving metabolic parameters and reducing the risk of type 2 diabetes, particularly among specific demographic groups. A study by Wang et al. sought to investigate the link between minerals and obesity in children, using data from the NHANES database from 2007-2014. This cross-sectional study aimed to enhance understanding of the role of minerals in preventing and managing obesity in children at high risk (31).

The existing research and reviews underscore the critical role of minerals in metabolic health and the need for comprehensive studies to further understand their impact on metabolic diseases, including obesity, metabolic syndrome, and diabetes. The findings emphasize the importance of monitoring mineral intake, addressing potential imbalances, and conducting further research to advance our understanding of mineral metabolism in the context of metabolic disorders (32, 33).

The study by Zhang et al. investigated the impact of iron overload on the development of non-alcoholic fatty liver disease (NAFLD). The research suggested that introducing iron to male Sprague Dawley rats fed a high-fat diet could synergistically exacerbate lipid metabolism disorders, liver injury, and oxidative damage. Additionally, treating the rats with deferoxamine (DFO) indicated potential support in reducing lipid metabolism dysfunction and the progression of NAFLD. The role of iron in chronic liver disease has been a subject of interest, as iron accumulation has been observed in various liver conditions, including hereditary hemochromatosis, alcoholic liver disease, nonalcoholic fatty liver disease, and hepatitis C viral infection. Higher iron levels are present not only in patients with hereditary hemochromatosis but also in those with acquired metabolic disorders and viral infections. Iron regulation may be disrupted in patients with chronic liver disease, as the liver’s synthetic functions, including the production of hepcidin, are decreased (34). Iron overload can lead to hepatic inflammation and alter lipid metabolism, contributing to the pathophysiology of chronic liver disease. The accumulation of iron in the liver is a common occurrence in conditions such as hereditary hemochromatosis, alcoholic liver disease, and nonalcoholic fatty liver disease. Ding et al. conducted a systematic review and meta-analysis of observational studies to investigate the associations of dietary iron, copper, and selenium levels with metabolic syndrome. The results of the study suggested a positive association between dietary iron levels and metabolic syndrome, while a negative association was found between dietary copper and selenium levels and metabolic syndrome.

Zaborova et al. conducted a study to investigate the levels of macro- and microelements, including potassium, rubidium, magnesium, calcium, and cesium, in the bodies of young adult athletes. The researchers aimed to understand the mineral levels in athletes during physical activity. The study found that wrestlers had higher levels of several macro- and microelements, including some toxic ones. The research highlights the significance of monitoring mineral levels in athletes to support their overall health and performance.

The articles published in this Research Topic emphasize the significance of minerals, their levels, their interrelationships, and their role in maintaining metabolic homeostasis. Experimental and clinical studies have revealed a connection between mineral intake, concentration in the body, and the incidence of metabolic disorders. The involvement of minerals in muscle contraction, heart rhythm, nerve impulse conduction, oxygen transport, enzyme activation, immune functions, antioxidant activity, and bone health underscores their essential role in the human body.

The articles in this Research Topic also serve to raise awareness and enhance the knowledge of healthcare providers regarding the importance of maintaining appropriate mineral levels to support metabolic homeostasis and prevent or delay the onset of diseases. Collectively, this body of research underscores the necessity for dialogue within the healthcare community to further explore the role of minerals in the prevention and management of metabolic diseases. Additionally, it emphasizes the importance of implementing effective screening and monitoring of mineral levels during routine physical examinations to increase awareness and encourage the consumption of healthier foods that are rich in essential minerals (35).

## Author contributions

M-LH: Writing – original draft, Writing – review & editing. MR: Writing – original draft, Writing – review & editing.
